# Endometrial Carcinoma in a 26-Year-Old Obese Woman With Polycystic Ovary Syndrome: A Case Report

**DOI:** 10.7759/cureus.89612

**Published:** 2025-08-08

**Authors:** Bubli Ahmed, Rabeya Chowdhury, Syeda A Shirin, Al H Habib

**Affiliations:** 1 Obstetrics and Gynecology, Jalalabad Ragib Rabeya Medical College, Sylhet, BGD; 2 Obstetrics and Gynecology, Flushing Hospital Medical Center, Flushing, USA; 3 Obstetrics and Gynecology, Dhaka Medical College, Dhaka, BGD; 4 Internal Medicine, Khwaja Yunus Ali Medical College, Sirajganj, BGD

**Keywords:** endometrial cancer, fertility, hypertension, pcos, young age

## Abstract

Endometrial carcinoma is one of the most common gynecologic cancers worldwide. The condition typically occurs after menopause; however, young women under the age of 40 years can also be diagnosed with the disease. Providers may delay diagnosis in young patients due to nonspecific presentation or low clinical suspicion. Standard treatment includes surgery, chemotherapy, and adjuvant radiotherapy. All these therapeutic interventions are determined by the patient's tumor stage and prognosis. However, in the case of a young patient, treatment options are determined by their desire to maintain fertility and ovarian function. We reported a case of a 26-year-old woman who presented to the gynecological office complaining of irregular menstrual cycles and oligomenorrhea. Endometrioid endometrial carcinoma was found after diagnostic hysteroscopy, followed by dilation and curettage (D&C) and a tissue examination. Our patient desired a surgical option as her family was complete. This case illustrates the importance of early diagnostic evaluation in young women with risk factors and highlights individualized decision-making in treatment planning.

## Introduction

Endometrial carcinoma is a type of cancer that develops in the lining of the uterus. In the United States, it is the fourth most common cancer. Studies showed that, from 2012 to 2021, the incidence rose by 0.6% per year among white women and 2% to 3% among all other racial and ethnic groups [1,2]. The mortality rate of uterine cancer increased by 1.5% per year from 2013 to 2022 [2,3]. The average age of diagnosis is around 60 years, and it is uncommon in individuals under the age of 40. Endometrial carcinoma in young women under 40 accounts for approximately 5% of cases [4]. For that reason, delayed diagnosis can occur due to nonspecific symptoms and a low index of suspicion. Recognizing the condition early in this population is vital, particularly when fertility preservation is a consideration.

## Case presentation

**Figure 1:**
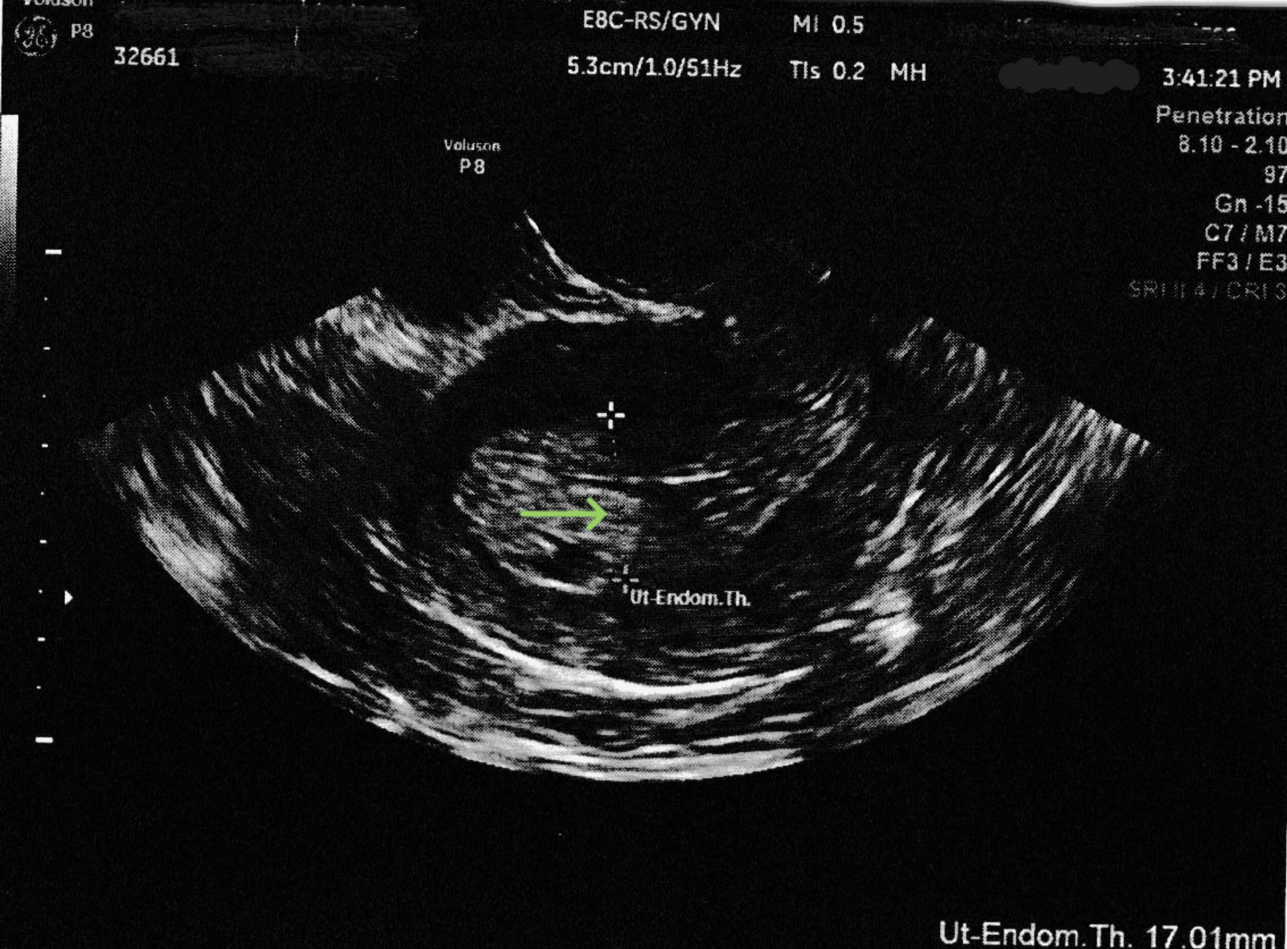
Pelvic transvaginal ultrasound showing an endometrial thickness of 17 mm

**Figure 2:**
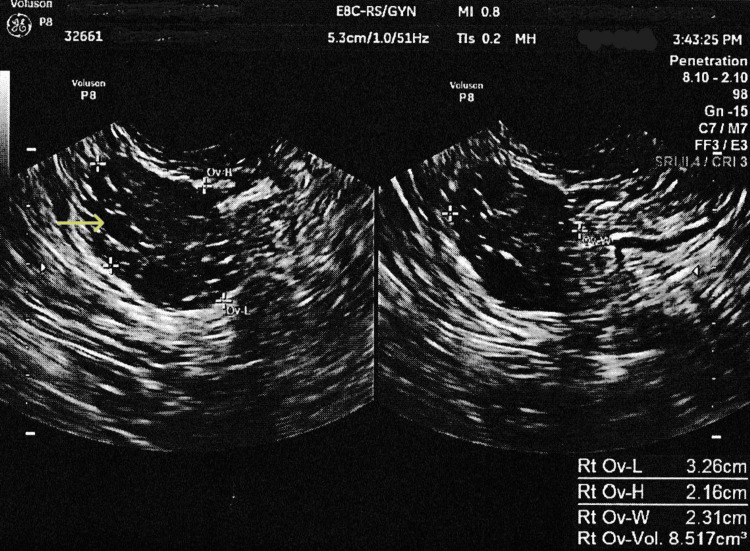
Pelvic transvaginal ultrasound showing a polycystic right ovary

A 26-year-old female diagnosed with polycystic ovary syndrome (PCOS), who is gravida 2 and para 2002 (indicating two term deliveries and two living children), presented with complaints of an irregular menstrual cycle and oligomenorrhea. Her irregular menstruation started early in her life, cycles every 45-60 days, lasting 7-10 days. The patient denied any postcoital bleeding, pelvic pain, or discharge. Her BMI was elevated at 46.59 kg/m^2^. She has had chronic hypertension, which was well controlled with labetalol 100 mg twice daily. A pelvic transvaginal ultrasound (TVS) showed that the lining of her uterus was thick and heterogenous, measuring 17.0 mm, with a possible polyp that was 1.6 x 1.3 cm in size. The right ovary showed polycystic morphology (Figures 1, 2).

Based on TVS findings and consideration of increased estrogen exposure due to obesity and PCOS, she was selected as a candidate for hysteroscopic dilation and curettage (D&C). The patient was thought to have hyperplasia or endometrial carcinoma based on the polypoid appearance of the endometrium under the hysteroscope. Hysteroscopic polypectomy and curettage were performed, and the specimen was then sent for histopathology. The pathology report showed well-differentiated endometrioid (International Federation of Gynecology and Obstetrics [FIGO] grade 1) adenocarcinoma with proliferative endometrium. Immunohistochemical staining for MLH-1, PMS-2, MSH-2, and MSH6 showed intact nuclear staining in the tumor (no loss of staining). These suggested a low probability of Lynch syndrome or sporadic MSI-high tumor. We referred the case to gynecologic oncology, where a pelvic MRI was performed to determine the staging of the disease. The result of the pelvic MRI revealed carcinoma confined to the endometrium only, and other pelvic organs were normal. Finally, she underwent a hysterectomy without bilateral salpingo-oophorectomy. Her Pap smear was negative for intraepithelial lesion or malignancy. Her other lab tests were as follows: HbA1C 5.6, follicle-stimulating hormone 5.7 mIU/mL, luteinizing hormone 6.3 mIU/mL, prolactin 7.7 ng/mL, and thyroid-stimulating hormone 1.74 mIU/L (Table 1).

**Table 1:** Lab results. FSH, follicle-stimulating hormone; LH, luteinizing hormone; TSH, thyroid-stimulating hormone

Test	Patient Value	Reference (Normal) Range	Units	Interpretation
Hemoglobin A1c	5.6	<5.7%	%	Normal (no diabetes)
FSH	5.7	Follicular: 2.5–10.2; mid-cycle: 3.1–17.7; luteal: 1.5–9.1; postmenopausal: 23.0–116.3	mIU/mL	Normal (reproductive age)
LH	6.3	Follicular: 1.9–12.5; mid-cycle: 8.7–76.3; luteal: 0.5–16.9; postmenopausal: 10.0–54.7	mIU/mL	Normal (likely follicular or luteal phase)
Prolactin	7.7	Non-pregnant: 3.0–30.0; pregnant: 10.0–209.0; postmenopausal: 2.0–20.0	ng/mL	Normal (non-pregnant)
TSH	1.74	≥20 years: 0.40–4.50	mIU/L	Normal thyroid function

## Discussion

Most patients with endometrial carcinoma present with abnormal uterine or vaginal bleeding, including postmenopausal bleeding. In addition, symptoms may include lower abdominal pain, pelvic pain, dyspareunia, dysuria, or vaginal discharge. Our patient presented with oligomenorrhea - a less common symptom that can lead to delayed diagnosis in younger women due to a lower index of clinical suspicion. In this age group, such symptoms are often attributed to benign conditions such as hormonal imbalance, PCOS, endometrial hyperplasia, and uterine or endocervical polyps. At this young age, the diagnosis of endometrial carcinoma is rare, making this case particularly noteworthy for its diagnostic process and favorable outcome. We approached the case with careful history-taking, identification of risk factors, and a thorough diagnostic workup, including exclusion of more common differential diagnoses.

Initial evaluation includes transvaginal and transabdominal ultrasound to assess the endometrium. Postmenopausal women need tissue sampling if their endometrial thickness exceeds 5 mm or 8 mm for those on hormonal therapy. In premenopausal women, endometrial thickness varies with the menstrual cycle but should not exceed 16 mm [4]. If the endometrial lining is unclear, other ultrasound signs, such as an uneven or irregular endometrium, along with a mass or fluid buildup, should be checked. A TVS can be repeated 10 to 14 days later after the initial ultrasound in different phases of the menstrual cycle. In our case, TVS revealed heterogeneous endometrium with a thickness of 17.0 mm.

There are different subtypes of endometrial carcinoma. Type I endometrial carcinoma is called endometrioid endometrial carcinoma, which represents 80% of endometrial carcinoma and is usually associated with high estrogen exposure, mostly affecting patients aged 55 to 65 years. Type II endometrial carcinoma is known as non-endometrioid endometrial carcinoma, which accounts for 20% of cases. The causes are usually endometrial atrophy. Type II disease typically affects women aged 65 to 75 years.

Our patient was diagnosed with type I endometrial carcinoma. The risk factors of endometrial carcinoma in young women are obesity, smoking, PCOS, insulin resistance, type 2 diabetes, hypertension, and early menarche. Higher BMI is a significant risk factor for endometrial cancer in young women, with a 22-fold increased risk in women with a BMI over 35 [5]. In addition, endometrial carcinoma is also associated with Lynch syndrome. The risk factors in our patient were PCOS, obesity, and hypertension. She had not undergone any treatment for PCOS.

Any premenopausal woman with prolonged or heavy and irregular menstrual bleeding, especially with risk factors, should undergo thorough evaluation, including pelvic imaging and, if indicated, histopathologic assessment. The treatment of endometrial carcinoma is based on FIGO staging. The surgical management is hysterectomy with bilateral salpingo-oophorectomy, with or without lymphadenectomy [5]. Adjuvant chemotherapy and radiotherapy may be required. Our patient underwent a laparoscopic total hysterectomy without bilateral salpingo-oophorectomy under general anesthesia. Intraoperative findings showed no visible evidence of extrauterine disease. Surgical intervention was selected due to patient preference, as her family was complete, and ovarian preservation was considered because the cancer is low risk, the patient is young, and there is no evidence of spread beyond the uterus.

Before surgery, a further radiological assessment should be performed, which includes a contrast CT or MRI scan to detect metastases. An MRI of the pelvis is important to find out how far the cancer has spread, especially to see if it has invaded the cervical stroma and myometrium. MRI is preferred over a CT scan for staging purposes, as it gives a clear visualization of myometrial invasion. We use nuclear imaging methods such as PET or CT to identify distant metastases. Synchronous ovarian cancer is more common in women under 40 compared to the age group of 41 to 60 [6]. Our patient did not present with synchronous ovarian cancer. Young patients diagnosed with low-grade tumors at an early stage typically have an excellent prognosis. Most of the women of reproductive age opt for fertility-preservation options [5]. Conservative treatment strategies should be pursued to ensure accurate cancer staging and detect any additional tumors that may be present simultaneously (synchronous) or develop later (metachronous). Before initiating conservative treatment, it is crucial to limit the cancer to the endometrium and maintain its low grade.

Patients with endometrial carcinoma are advised to maintain a healthy diet and lifestyle modifications, including a high-protein diet and exercise. Follow-up for endometrial cancer includes CT, MRI, and PET. The tumor marker CA-125 should be measured at diagnosis and monitored post-treatment to detect recurrence. In our case, CA-125 was not measured at the time of diagnosis, representing a limitation in baseline assessment. Approximately 4-20% of patients with endometrial cancer experience regional recurrence, with the highest rates occurring in those with locally advanced disease, typically within the first two years following treatment. Our patient followed up with oncology, where she was recognized as cancer-free at six months.

## Conclusions

This case underscores the importance of considering endometrial carcinoma in the differential diagnosis of abnormal uterine bleeding in young women, particularly those with risk factors such as PCOS, obesity, and hypertension. Early evaluation and timely diagnosis are critical to improving outcomes. Individualized treatment planning, including the consideration of fertility preservation, is essential in this population. Clinicians should maintain a high index of suspicion and approach such cases with comprehensive diagnostic assessment, even in patients of reproductive age, to avoid delays in diagnosis and ensure optimal care.
